# Corrigendum: Consistent phosphenes generated by electrical microstimulation of the visual thalamus. An experimental approach for thalamic visual neuroprostheses

**DOI:** 10.3389/fnins.2021.666602

**Published:** 2021-03-17

**Authors:** Fivos Panetsos, Abel Sanchez-Jimenez, Elena Rodrigo-Diaz, Idoia Diaz-Guemes, Francisco M. Sanchez

**Affiliations:** ^1^Neurocomputing and Neurorobotics Research Group, Complutense University of Madrid, Madrid, Spain; ^2^School of Optics, Complutense University of Madrid, Madrid, Spain; ^3^Faculty of Biology, Complutense University of Madrid, Madrid, Spain; ^4^Applied Research, “Jesus Uson” Minimally Invasive Surgery Centre, Caceres, Spain

**Keywords:** LGN, V1, phosphene, visual percept, BMI, implant

An author name was incorrectly spelled as Elena Diaz-de Cerio. The correct spelling is Elena Rodrigo-Diaz.

The authors apologize for this error and state that this does not change the scientific conclusions of the article in any way. The original article has been updated.

